# Transplantation of a vascularized pedicle of hemisected spinal cord to establish spinal cord continuity after removal of a segment of the thoracic spinal cord: A proof‐of‐principle study in dogs

**DOI:** 10.1111/cns.13696

**Published:** 2021-06-28

**Authors:** Shuai Ren, Weihua Zhang, HongMiao Liu, Xin Wang, Xiangchen Guan, Mingzhe Zhang, Jian Zhang, Qiong Wu, Yan Xue, Dan Wang, Yong Liu, Jianyu Liu, Xiaoping Ren

**Affiliations:** ^1^ Hand and Microsurgery Center The Second Affiliated Hospital of Harbin Medical University Harbin China; ^2^ State‐Province Key Laboratories of Biomedicine‐Pharmaceutics Harbin Medical University Harbin China; ^3^ Global Initiative to Cure Paralysis (GICUP) Columbus OH USA; ^4^ Department of Orthopedics Ruikang Hospital Affiliated to Guangxi University of Chinese Medicine Nanning China; ^5^ Institute of Orthopedic Ruikang Hospital Affiliated to Guangxi University of Chinese Medicine Nanning China; ^6^ Department of Pathology The General Hospital of Heilongjiang Farms & Land Reclamation Administration Harbin Harbin China; ^7^ Department of MR Diagnosis The Second Affiliated Hospital of Harbin Medical University Harbin China; ^8^ Department of Orthopaedics The Fifth Hospital of Harbin Harbin China

**Keywords:** GEMINI, polyethylene glycol, spinal cord, spinal cord fusion, spinal cord grafting, spinal cord injury

## Abstract

**Introduction:**

Glial scar formation impedes nerve regeneration/reinnervation after spinal cord injury (SCI); therefore, removal of scar tissue is essential for SCI treatment.

**Aims:**

To investigate whether removing a spinal cord and transplanting a vascularized pedicle of hemisected spinal cord from the spinal cord caudal to the transection can restore motor function, to aid in the treatment of future clinical spinal cord injuries. We developed a canine model. After removal of a 1‐cm segment of the thoracic (T10–T11) spinal cord in eight beagles, a vascularized pedicle of hemisected spinal cord from the first 1.5 cm of the spinal cord caudal to the transection (cut along the posterior median sulcus of the spinal cord) was transplanted to bridge the transected spinal cord in the presence of a fusogen (polyethylene glycol, PEG) in four of the eight dogs. We used various forms of imaging, electron microscopy, and histologic data to determine that after our transplantation of a vascular pedicled hemisection to bridge the transected spinal cord, electrical continuity across the spinal bridge was restored.

**Results:**

Motor function was restored following our transplantation, as confirmed by the re‐establishment of anatomic continuity along with interfacial axonal sprouting.

**Conclusion:**

Taken together, our findings suggest that SCI patients—who have previously been thought to have irreversible damage and/or paralysis—may be treated effectively with similar operative techniques to re‐establish electrical and functional continuity following SCI.

## Graphical abstract

After transplantation of a vascularized pedicle of hemisected spinal cord to bridge the transected spinal cord, various forms of imaging, electron microscopy, and histological data can confirm that electrical continuity across the spinal bridge was restored. Patients with SCI previously thought to have irreversible damage and/or paralysis can be treated effectively with similar operative techniques.

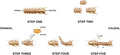

## 1 INTRODUCTION

Spinal cord injury (SCI) results in major issues for both individuals suffering from SCI and for society.^1^, ^2^, ^3^ Indeed, tens of thousands of people suffer from severe disabilities as a result of SCI, as well as multiple complications related to SCI. Apart from the direct impact of SCI on patients and their families, SCI also places a heavy burden on society, in terms of social and financial costs.^4^

Studies have shown that extensive damage occurs within 30 min of trauma, during which axons die back hundreds of micrometers. In addition, some in‐vivo imaging studies have demonstrated that axonal regeneration can occur within 6–24 h of the lesion.^5^, ^6^ Recent research has shown that there are two parallel cellular pathways from the brain to the spinal cord.^7^, ^8^ In addition to the pyramidal tract, there is a gray matter‐based network of interneurons extending from the brainstem to the spinal cord that is involved in the conduction of command signals from cortical motor areas to peripheral motor neurons. This short‐fiber pathway is known as the cortico‐truncoreticulo‐propriospinal pathway, which embeds and links the central pattern generators located in both the cervical and lumbar spinal cord.^7^, ^8^ After spinal cord injury, this short fiber regenerates much faster than the long fibers in the pyramidal tract. Thus, if two fresh and healthy transected spinal cord sections are created after spinal cord injury, and they can be fused quickly to rebuild the electrophysiological connection, further damage can be minimized, thereby increasing the patient's chances of healing.

Polyethylene glycol (PEG) is a water‐soluble polymer that is synthesized from ethylene oxide and has a molecular weight ranging from 0.4 to 100 kDa. Non‐toxic and non‐irritating, it is widely used in various pharmaceutical preparations. PEG has been approved by the U.S. Food and Drug Administration (FDA) and the China National Food and Drug Administration (CFDA) for clinical use. Extensive experimental evidence has shown that polyethylene glycol (PEG) can act as a neuroprotective agent to not only prevent apoptosis but also to seal damaged membranes and thereby promote membrane fusion, allowing for acute restoration of the integrity of sharply severed nerve fibers.^9^, ^10^, ^11^, ^12^, ^13^ Hence, when PEG is utilized after resection of a limited segment of spinal cord and re‐establishes apposition of the severed ends of the spinal cord, through the membrane fusion function of PEG, the spontaneous regrowth of severed axons/dendrites of the apposed spinal cord occurs at the contact point, re‐establishing the gray matter neurophil.^11^ As such, the bridged gray matter is then connected to the white matter on both sides, thereby restoring electrophysiological conduction.

We and others have shown this dramatic re‐establishment of electrical continuity in several animal models of complete spinal cord transection (mice, rats, dogs, pigs, and monkeys) using PEG as a neuroprotective agent at the site of transection. In these studies, all animals eventually recovered a large part of their motor functions.^10^, ^14^, ^15^, ^16^, ^17^, ^18^, ^19^ However, this experimental model does not recapitulate traumatic SCI in humans; in contrast, patients with paraplegia secondary to traumatic SCI have glial scars. Therefore, a surgical method that meets the following conditions needed to be designed: (1) The glial scar needs to be resected acutely, leaving the cranial and caudal ends of the otherwise normal spinal cord; (2) These two freshly transected sections need to be connected immediately without any tension or a gap. Given the above two conditions, there are three potential surgical methods, as follows: (1) transplantation of a stem cell‐based tissue engineering scaffold; (2) operative shortening of the vertebral column (by vertebrectomy or multiple discectomies), and approximation of the two freshly severed spinal cord stumps; and (3) autotransplantation of a vascularized segment of the caudal spinal cord to act as a neural bridge that will allow re‐establishment of electrical and functional continuity across the gap left after resection of the glial scar. In the present study, we employed autotransplantation since it circumvents immune‐related issues and allows for a vascularized spinal segment that can be mobilized to fit the SCI gap.

## 2 METHODS

### 2.1 Animals

All procedures dealing with animals have been ethically reviewed and approved by the Institutional Animal Care and Use Committee of Harbin Medical University (IACUC) and the Institute of Laboratory Animal Science of China (A5655‐01). These procedures were also in accordance with Directive 2010/63/EU of the European Parliament. Animal data reporting followed The ARRIVE 2.0 guidelines.^20^ In this study, in a large animal experiment preceding non‐human primate experiments, we chose to use 8‐month‐old female beagles weighing 8 kg. Because the beagle has a docile personality, few genetic diseases, and good experimental reproducibility, it is recognized by the biological community as an ideal experimental dog. Moreover, female beagles have stronger resistance to urinary tract infections than males, and young dogs have a better recovery ability than adults. Beagles were obtained from Qingdao Agricultural University and were housed comfortably with a 12/12‐h light/dark cycle and food and water were provided ad libitum. This experiment consisted of four dogs in the experimental group that underwent spinal transection and PEG treatment (TRANSPLANT+PEG), and four dogs in the control group that only underwent spinal transection (TRANSPLANT). At 6 months after spinal transections, all dogs were sacrificed (by overdose of anesthetics) after numerous assessments of motor function.

### 2.2 Spinal cord excision and autotransplantation

**FIGURE 1:**
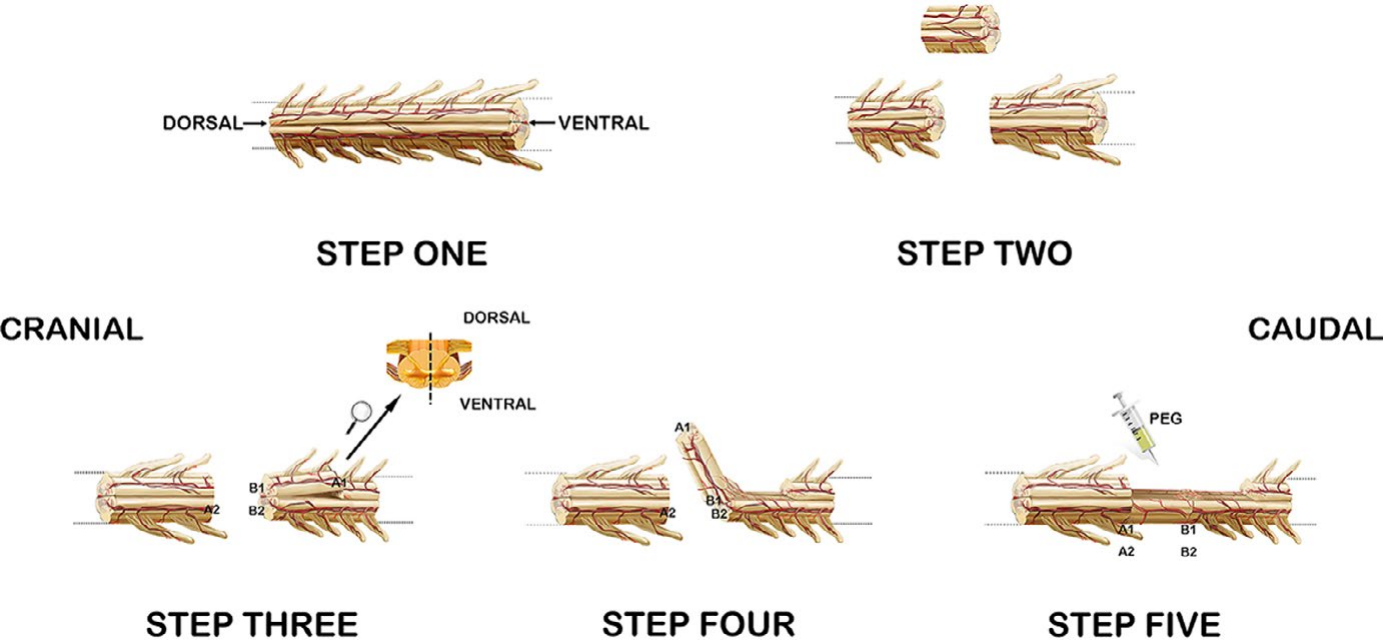
Surgery. STEP ONE: a dorsal view of the complete spinal cord is shown, with the left side being the cranial side and the right side being the caudal side. STEP TWO: complete removal of a 1‐cm‐long segment of the spinal cord via sharp dissection at the T10 segment of the spinal cord is shown. STEP THREE: a hemisegment of the distal spinal cord was cut along a cross‐section at a distance of 1.5 cm from the gap, after which we continued to cut along the posterior median sulcus of the spinal cord to the gap (the side close to the gap was not completely cut, so the blood supply to this half of the spinal cord was maintained). STEP FOUR: the hemisection was then mobilized to fill the gap left after removing the T10 section of the spinal cord. STEP FIVE: we then injected PEG at the transplant site. The half of the spinal cord that was recently flipped was tightly connected to the cranial face of the spinal cord and the remaining half of the caudal spinal cord

During the entire operation, each dog was kept in a prone position. Ketamine (0.1 mg/kg given intramuscularly) was administered to induce general anesthesia before intubation for artificial ventilation. Anesthesia was maintained through intravenous administration of remifentanil (0.2 μg/kg/min), vecuronium bromide (0.1 mg/kg), and propofol (10 mg/kg/h). An incision overlying the thoracic spinal column was made at T10–T11, and a laminectomy was conducted using standard neurosurgical techniques to expose the spinal cord. Then, 1 cm of the spinal cord at T10 was removed via sharp transection using a sapphire blade (Shanghai Jingming Fine Technology Co.) both cranially and caudally to create a gap, as described previously^7^ (Figure 1). Thereafter, the distal 1.5 cm of the caudal spinal cord was hemisectioned transversely, and the blood supply was preserved to the now mobilized hemisection to be used to bridge the gap from the excised spinal cord. Thereafter, random numbers were used to randomize dogs to either the experimental condition, where the autotransplantation site was bathed in PEG 600 (2 ml) for 5 min, or the control condition, where the autotransplantation site was bathed in 0.9% NaCl (2 ml) for 5 min (0.9% NaCl is an isotonic solution that will not damage cells). Standard closure by layers was subsequently performed.

For three consecutive days following the above procedures, all dogs were given an intravenous solution containing sulbactam sodium and cefoperazone (25 mg/kg; Harbin General Pharmaceutical Factory's Sales Company) Urine was removed from the bladder via abdominopelvic compressions twice a day throughout the period of observation of the study, or until the voiding reflex was restored. In the absence of recovery, most dogs resumed oral intake of a normal diet (Bridge PetCare Co., Ltd) by postoperative day 3. Dogs had free access to water or were hydrated intravenously with electrolyte solutions when they were unable to eat or drink during the first postoperative week. According to the criteria of the Appropriate Care and Use of Laboratory Animals of the National Research Council (Washington, D.C., USA),^21^ the SCI dogs were screened twice a day for 30 min each time for signs of dehydration, infection, and other possible complications. The dogs were able to move as their upper body was functional, although there were some difficulties due to the complete paralysis of their lower extremities. Four times a day, each SCI dog underwent lower limb rehabilitation using range‐of‐motion exercises and hip massages. The dogs were also supported in a custom‐made, wheelchair‐like support that allowed mobilization until useful voluntary motor function was regained; this support also helped to alleviate or prevent pressure sores and skin irritations. All eight dogs survived the operation and completed the study without obvious discomfort.

### 2.3 Motor assessments

Motor function was assessed 12 times after the operation at 3, 10, 17, 21, 25, 29, 32, 37, 45, 53, 59, and 180 days postoperatively. Specifically, multiple aspects of motor function and mobility were evaluated, including stability, trunk position, coordination, and open‐field mobility. At each time point, the canine Basso‐Beattie‐Bresnahan rating scale (cBBB) was used to assess motor function on a 20‐point scale by two trained reviewers blinded to the experimental group in which each dog was allocated. A cBBB score of 0 signifies total paraplegia, while a cBBB score of 19 signifies normal functioning.^22^ Statistical significance at each time point was calculated using two‐way analysis of variance and Mann–Whitney *U*‐test.

### 2.4 Neuroimaging

Magnetic resonance imaging (MRI) and diffusion tensor imaging (DTI) were conducted on all dogs in the prone position using a 3.0‐T MRI system (Achieva 3, Philips) At 180 days post‐operation, sagittal, T2‐weighted, fast spin‐echo sequences were acquired with the following parameters: TR = 1700 ms; TE = 100 ms; slice thickness = 3 mm; slice gap = 0.1; and NSA = 4. Additionally, axial single‐shot echo‐planar DTI sequences were acquired with the following parameters: TR = 6100 ms; TE = 93 ms; voxel size = 2 mm × 2 mm; slice thickness = 2 mm; slice gap = 0; NSA = 2; and diffusion direction number = 15.

### 2.5 Tissue preparation

**FIGURE 2:**
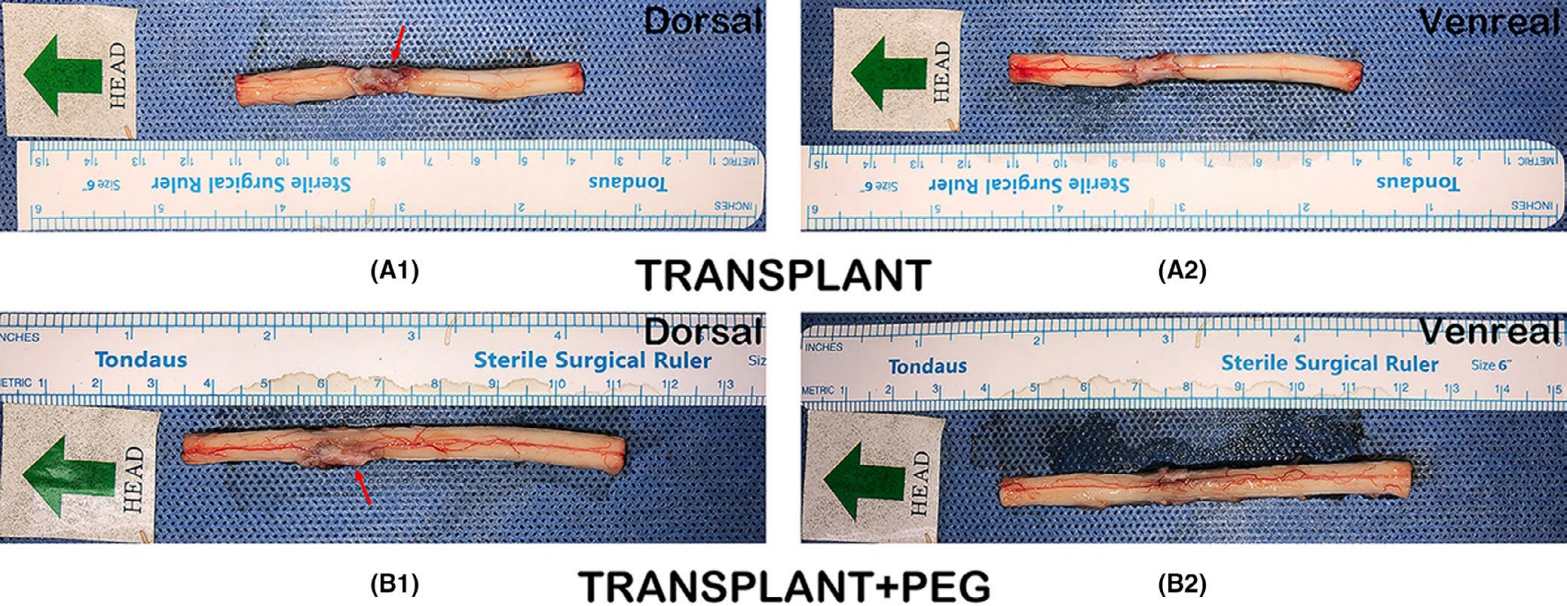
Spinal cord sample. A1 (dorsal) and A2 (ventral) show images from the TRANSPLANT group. It can be seen that there is a glial scar in A1, as indicated by the red arrow. B1 (dorsal) and B2 (ventral) show images of the TRANSPLANT+PEG group. The shape of the transplanted spinal cord was basically the same as that of the normal spinal cord. However, the other half of the gap consisted of a glial scar (red arrow in B1)

At 6 months after operations, all dogs were fully anesthetized with ketamine (0.1 mg/kg given intramuscularly) and perfused with 0.9% NaCl solution and 4% paraformaldehyde. Immediately thereafter, the thoracic spinal cord was extracted en bloc by sectioning 2 cm above and below the point of fusion (Figure 2). Subsequently, a small section (approximately 2 mm × 3 mm) was cut from the transplant area for electron microscopic observations. The remaining samples were used for histopathology and were immersed in 4% paraformaldehyde for 48 h, followed by paraffin embedding.

### 2.6 Transmission electron microscopy

At the deep site of the transplant area, we cut small blocks of approximately 1 mm^3^ and soaked them in 2.5% glutaraldehyde at 4°C for 24 h. The tissue blocks were washed three times in phosphate‐buffered saline (PBS) for 15 min. After fixation in 1% osmic acid for 1 h, 1% uranyl acetate was used to stain the tissues for 2 h. The tissues were then embedded for coronal sections after using gradient acetone solution for dehydration. Following localization by toluidine blue staining and semi‐thin sectioning, the tissues were observed using a transmission electron microscope (H‐7700, Hitachi) after being cut into ultrathin sections.

### 2.7 Scanning electron microscopy

At the deep site of the transplant area, we cut small blocks of approximately 1 mm^3^. These blocks were fixed in a solution containing 2% glutaraldehyde in 0.1 M of sodium cacodylate buffer at 4°C for 12 h. Thereafter, the tissue blocks were washed three times with 0.2 M of sodium cacodylate buffer and were then post‐fixed in a 1% osmium tetroxide solution for 1 h. Subsequently, they were dehydrated and mounted on aluminum stubs. A MED020 sputter coater (Balzers) was used to sputter coat the samples with copper, and they were then examined using a Hitachi 10‐kV scanning electron microscope (S‐3400N, Hitachi).

### 2.8 Standard histology

Sagittal sections (involving the entire cross‐section of the transplanted hemisegment of the spinal cord) at a thickness of 7 μm each were prepared from the paraffin‐embedded spinal cords and were stained with either hematoxylin‐eosin (HE), luxol fast blue (LFB), or 1% cresyl violet (Nissl staining). For HE staining, the 7‐μm‐thick sagittal sections were placed directly into xylene twice for 10 min for dewaxing, before being placed into alcohol at decreasing concentrations (100%, 95%, 85%, and 70%) for 5 min each for hydration. PBS was used to rinse the sections three times (5 min each) before they were stained with hematoxylin for 10 min and subsequently rinsed with distilled water. Finally, a 1% HCl solution was used to separate the cytoplasm from pigment; then, the sections were dipped in distilled water. Sections were stained with eosin for 3 min before dehydrating them with alcohol at increasing concentrations (70%, 85%, 95%, and 100%) for 5 min each. Thereafter, the sections were washed twice with xylene (5 min each) for vitrification.

For Nissl and LFB staining, the 7‐μm‐thick sections were dewaxed and hydrated, after which they were submerged in xylene for 20 min, 100% alcohol for 1 min, and 95% alcohol for 5 min. Nissl and LFB were then used to stain these sections for 10 min, followed by a triple rinse in 0.2% glacial acetic acid and counterstaining for 10 min in 0.5% brilliant‐green glacial acetic acid solution. Next, distilled water was used to rinse the sections for 2 min, prior to them being dehydrated in gradient ethanol and incubated twice in xylene (5 min each).

An Olympus IX73 optical microscope was used to visualize all sections. For image analysis, Image‐Pro‐Plus (Media Cybernetics) software was utilized.

### 2.9 Immunohistochemistry

The spinal cord tissues of the TRANSPLANT+PEG group and the TRANSPLANT group were cut into 10‐μm longitudinal sections, while the bridging tissue of the spinal cord of one beagle in the TRANSPLANT+PEG group was cut into 10‐μm coronal sections. The sections were then mounted onto silanized glass slides. There was considerable vacuolization of the bridging tissue of the spinal cord of the TRANSPLANT group, as shown by the HE staining results (Figure 7, red arrows in A). The coronal slices were difficult to mount onto the silanized glass slides, and therefore the TRANSPLANT group does not have coronal slices. After deparaffinization, the sections were quenched of endogenous peroxidase activity in 3% methanol hydrogen peroxide for 30 min. Employing the Abcam IHC fixation protocol (https://www.abcam.com/protocols/ihc‐fixation‐protocol), the antigen was then fixed in a citrate buffer (pH 6.0) via a microwave for 5 min. The sections were incubated with primary antibodies for neurofilament heavy polypeptide (1:50, Abcam) and myelin basic protein (MBP; 1:50, Abcam) overnight at 4°C. Thereafter, sections were incubated with a secondary antibody (PV secondary antibody kit, Beijing Zhongshan Jinqiao Biotechnology Co., Ltd) in the dark for 20 min at 37°C. Next, 3′‐diaminobenzidine (DAB) was added dropwise. Finally, the slides were mounted with a coverslip using VECTASHIELD (Vector Laboratories) for microscopic observation after hematoxylin counterstaining, dehydration, and clearing in xylene. The average integral optical density (AIOD) of areas positive for neurofilament (NF) and MBP was analyzed by Image‐Pro‐Plus 6.0 (Media Cybernetics). The degree of staining was reflected by the AIOD which, in turn, reflected the relative content of the examined substance, via the following formula: AIOD = IOD/*A*, where IOD is the integral optical density (IOD) and *A* is the total substance‐positive cell area.

### 2.10 Immunofluorescence

#### 2.10.1 Neurofilament and glial fibrillary acidic protein (GFAP)

The spinal cord tissues of the TRANSPLANT+PEG group and the TRANSPLANT group were cut into 10‐μm longitudinal sections, while the bridging tissue of the spinal cord of one beagle in the TRANSPLANT+PEG group was cut into 10‐μm coronal sections. The sections were then mounted onto silanized glass slides. Slides were air‐dried for 15 min and washed in PBS before blocking in 10% normal bovine serum and 0.1% Triton X‐100 in PBS at room temperature. Primary antibodies diluted in Dako antibody diluent were applied overnight in a humidified chamber at 4°C. Primary antibodies included anti‐neurofilament heavy polypeptide (1:100, Abcam) and anti‐glial fibrillary acidic protein (GFAP; 1:1000, Abcam). Slides were then washed and secondary antibodies (IgG‐H&L, Alexa Fluor 594; 1:500, Abcam) that were diluted in PBS were applied for 45 min at room temperature. This procedure was repeated again for a duration of 30 min using IgG‐H&L, Alexa Fluor 488 (1:500, Abcam). Finally, the slides were washed in PBS prior to being mounted with a coverslip using VECTASHIELD (Vector Laboratories).

#### 2.10.2 MBP and GFAP

Similar to the procedure for neurofilament and GFAP, 10‐μm sections of spinal cord tissues were mounted onto silanized glass slides, which were then air‐dried for 15 min and washed in PBS before blocking in 10% normal bovine serum and 0.1% Triton X‐100 in PBS at room temperature. Primary antibodies diluted in Dako antibody diluent were applied overnight in a humidified chamber at 4°C. Primary antibodies included anti‐GFAP (1:1000, Abcam) and anti‐MBP (1:100, Abcam). Slides were washed and then incubated with secondary antibodies (IgG‐H&L Alexa Fluor, 554; 1:1000, Abcam) diluted in PBS at room temperature for 1 h. Slides were washed again and then incubated with secondary antibodies (IgG‐H&L, Alexa Fluor 488; 1:500, Abcam) diluted in PBS for 30 min at room temperature. Finally, slides were washed in PBS and mounted with a coverslip using VECTASHIELD (Vector Laboratories). Images were obtained via a fluorescent microscope fitted with a digital camera system (Olympus IX73). Identical settings for amplifier gain, cutoff, and fluorescent exposure were ensured for all images.

### 2.11 Statistical analysis

Motor assessments (cBBB scores) were analyzed by two‐way analysis of variance (*α* = 0.05) and Mann–Whitney *U*‐test.

Histologic indices (the AIOD of neurofilament‐positive and MBP‐positive areas) were analyzed via Image‐Pro‐Plus 6.0 (Media Cybernetics).

## 3 RESULTS

### 3.1 Motor function is partially restored proved by motor assessment

Normal cBBB scores were observed initially for all eight dogs (Table 1). All dogs survived the operation and as expected, complete paraplegia was observed in each dog postoperatively (cBBB score of 0).

**TABLE 1:** Mann–Whitney *U*‐test shows there is a statistically significant difference between the two groups after the 29th day (*p* < 0.05)

cBBB														
Days	Transplant+PEG				Mean	Median	Transplant				Mean	Median	*U* value	*p* value
−1	19	19	19	19	19	19	19	19	19	19	19	19		
0	0	0	0	0	0	0	0	0	0	0	0	0		
3	0	0	0	0	0	0	0	0	0	0	0	0		
10	0	0	0	0	0	0	0	0	0	0	0	0		
17	0	0	1	1	0.5	0.5	0	0	0	0	0	0	4	0.343
21	0	0	3	4	1.75	1.5	0	0	1	0	0.25	0	5	0.486
25	1	1	4	6	3	2.5	0	0	1	1	0.5	0.5	2	0.114
29	4	4	5	6	4.75	4.5	1	2	1	1	1.25	1	0	0.029
32	7	6	6	6	6.25	6	2	2	2	1	1.75	2	0	0.029
37	9	8	7	9	8.25	8.5	2	2	2	1	1.75	2	0	0.029
45	10	8	9	10	9.25	9.5	2	2	2	1	1.75	2	0	0.029
53	12	8	10	11	10.25	10.5	2	3	2	2	2.25	2	0	0.029
59	12	9	10	12	10.75	11	2	3	3	2	2.5	2.5	0	0.029
180	12	11	10	12	11.25	11.5	4	4	5	3	4	4	0	0.029

**FIGURE 3:**
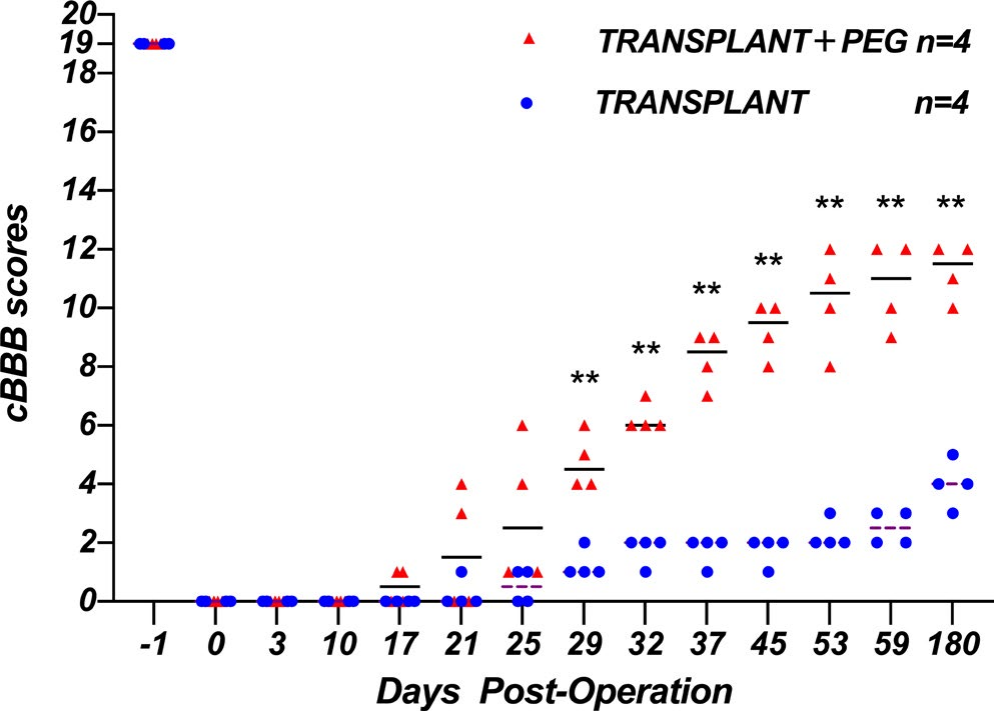
cBBB scores. cBBB scores were significantly improved in the TRANSPLANT+PEG group compared to those in the TRANSPLANT group at 29‐days post‐operation onward (Mann–Whitney *U*‐test, *p* < 0.05; the horizontal line represents the median)

Dogs in the TRANSPLANT+PEG group showed the first signs of motor recovery (autonomous hindlimb movement) within 17 days post‐operation, whereas the TRANSPLANT group showed some signs of recovery at 21 days post‐operation. Moreover, the subsequent improvement in the TRANSPLANT group was much more limited in comparison with that of the TRANSPLANT+PEG group. At 6 months post‐operation, all dogs in the TRANSPLANT+PEG group, but none in the TRANSPLANT group, regained the ability to walk autonomously (Figure 3). Two‐way analysis of variance showed a statistically significant difference between the TRANSPLANT+PEG group and the TRANSPLANT group (*F* = 329.8, *p* < 0.0001), and there were also statistically significant differences between different points in time (*F* = 109.5, *p* < 0.0001) (Table 2,a). We performed comparisons between groups at different time points, and the results are shown in Table 1. The Mann–Whitney *U*‐test indicated a statistically significant difference between the two groups after the 29th day (*p* < 0.05), and the mean value of the TRANSPLANT+PEG group was higher than that of the TRANSPLANT group.

**TABLE 2:** (a) Two‐way analysis of variance shows that there was a statistically significant difference between the TRANSPLANT+PEG group and the TRANSPLANT group (*F* = 329.8, *p* < 0.0001), and there were also statistically significant differences across different time points (*F* = 109.5, *p* < 0.0001). (b) Through the mixed‐effects analysis of the TRANSECTION+PEG group and the TRANSPLANT+PEG group, the *p* value and *F* value of the factor ‘time’ are <0.0001 and 114.6, respectively; thus, time has an impact on the recovery of spinal cord injury. The *p* value and *F* value of the factor ‘treatment (TRANSPLANT+PEG group or TRANSECTION+PEG group)’ were 0.6312 and 0.2469, respectively, so there was no significant difference between the TRANSPLANT+PEG group and the TRANSECTION+PEG group

(a) Two‐way ANOVA (TRANSPLANT+PEG group vs. TRANSPLANT group)					
	SS	DF	MS	*F* (DFn, DFd)	*p* value
Time	2635	13	202.7	*F* (2.301, 13.80) = 329.8	<0.0001
Treatment	357.1	1	357.1	*F* (1, 6) = 109.5	<0.0001

(b) Mixed‐effects analysis (TRANSPLANT+PEG group vs. TRANSECTION+PEG group)				
	*p* value		Statistically significant (*p* < 0.05)?	*F* (DFn, DFd)	
Time	<0.0001		Yes	*F* (1.531, 13.67) = 114.6	
Treatment	0.6312		No	*F* (1, 9) = 0.2469	

### 3.2 **Neuroimaging shows that nerve fiber** tracts **pass through the graft and restore continuity**

**FIGURE 4:**
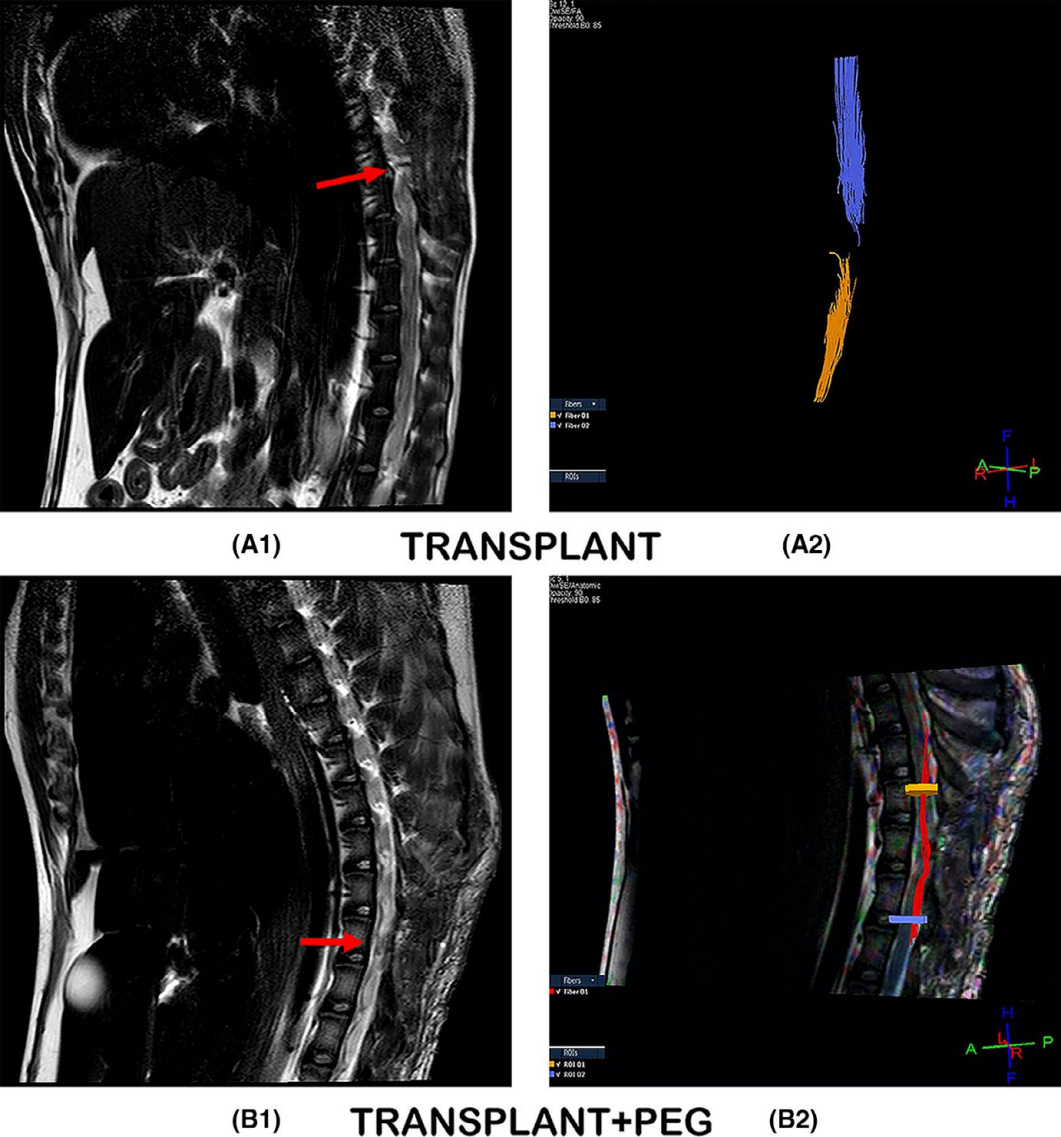
T2W and DTI. T2‐weighted MRI scans showing tissue re‐establishment of anatomic continuity in the TRANSPLANT+PEG group (red arrow in B1) in contrast to no such continuity in the TRANSPLANT group (red arrow in A1). DTI, which allows for the measurement of the integrity of nerve fiber tracts, showed fiber regrowth across the plane of transection in the TRANSPLANT+PEG group (B2), versus no such regrowth in the TRANSPLANT group (A2)

Due to poor image stability during live DTI examinations (due to inadequate anesthesia, each dog was not completely still during DTI, and body shaking caused artifacts in the DTI results), we only have 6 months of image data available. DTI can show the shape of the white matter nerve fiber bundles in the spinal cord. If the white matter nerve fiber bundles in the spinal cord are continuous (Figure 4, B2), the continuity can be shown in the DTI image; otherwise, the white matter nerve fibers will be displayed. Beam break is shown in Figure 4, A2. On visual inspection, T2‐weighted MRI scans showed increased re‐establishment of anatomic tissue continuity across the site of the graft in the TRANSPLANT+PEG group, as compared to that of the TRANSPLANT group (Figure 4, A1 and B1). By measuring the integrity of nerve fiber tracts, DTI results demonstrated fiber regrowth across the plane of transection in the TRANSPLANT+PEG group. In contrast, fiber regrowth was not evident in the TRANSPLANT group (Figure 4, A2 and B2).

### 3.3 A large amount of new myelin and axons found by transmission electron microscopy

**FIGURE 5:**
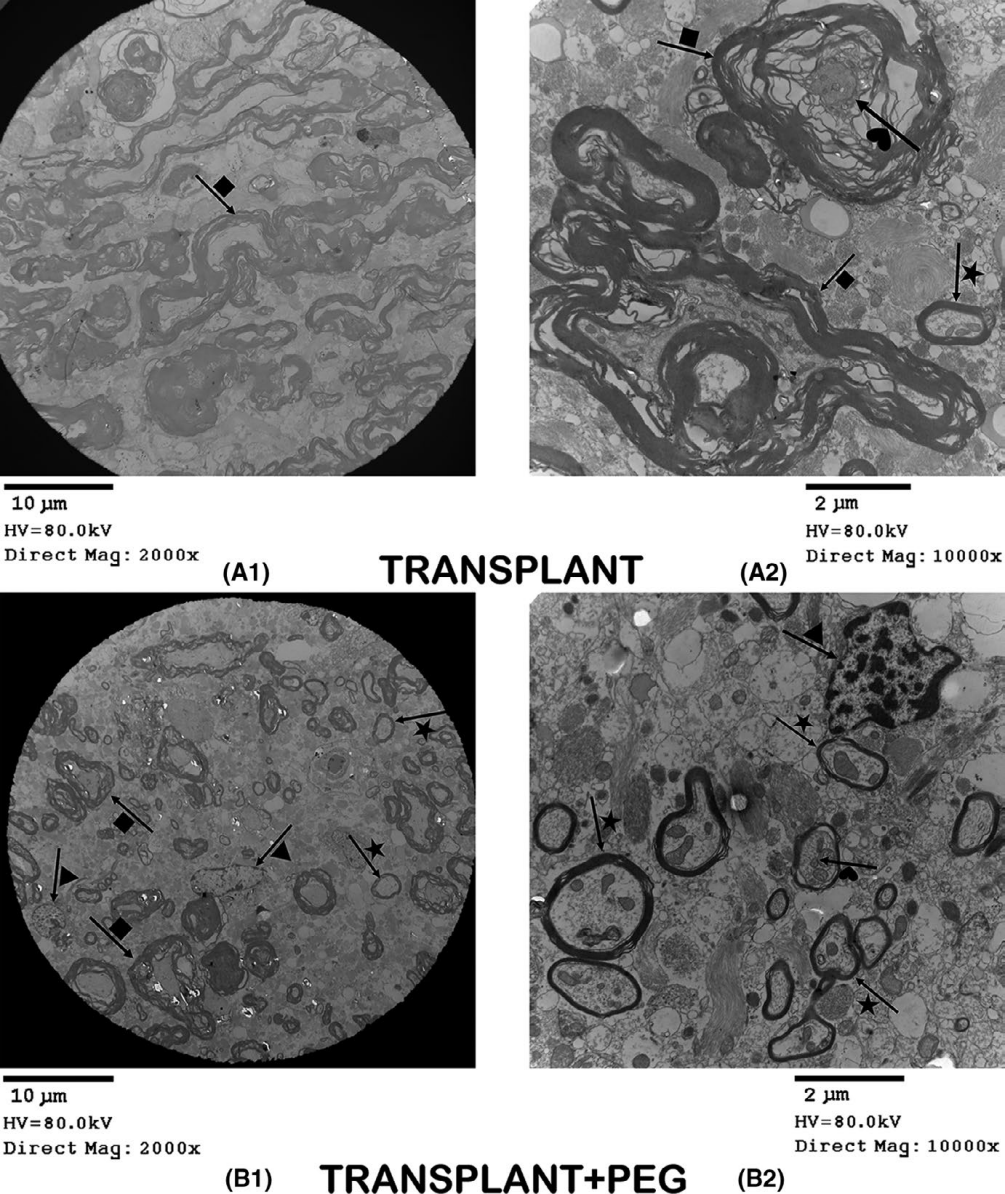
Transmission electron microscopy. In the TRANSPLANT+PEG group, we can see a large amount of new myelin (arrow + star in B1 and B2) and a small number of demyelinated axons (arrow + square in B1). In contrast, in the TRANSPLANT group, there is a lot of demyelinated myelin (arrow + square in A1 and A2), but less new myelin (arrow + star in A2). In the TRANSPLANT group, we see only few oligodendrocytes; while in the TRANSPLANT+PEG group, there are a large number of oligodendrocytes (arrow + triangle in B1 and B2). There is a clear contrast between the axons of the two groups: the axons of the TRANSPLANT group are atrophied (arrow + heart in A2), while those of the TRANSPLANT+PEG group are healthy (arrow + heart in B2)

Prior research has shown that axons undergo demyelination and necrosis secondary to Wallerian degeneration in the distal portion of severed axons when cytologically disconnected from their cell bodies. SCI can produce varying severities of axonal degeneration and necrosis,^5^, ^23^, ^24^, ^25^ which determine the extent of recovery from SCI.^26^ In the TRANSPLANT+PEG group, there was a large amount of new myelin (Figure 5, arrow + star in B1 and B2) and only a small amount of demyelinated myelin at the site of spinal cord fusion (Figure 5, arrow + square in B1). In contrast, in the TRANSPLANT group, larger quantities and degrees of demyelination were present (Figure 5, arrow + square in A1 and A2), with less new myelin (Figure 5, arrow + star in A2). Myelin in the CNS is composed of oligodendrocytes. In the TRANSPLANT group, oligodendrocytes were rarely seen; in contrast, in the TRANSPLANT+PEG group, a large number of oligodendrocytes were present (Figure 5, arrow + triangle in B1 and B2). There was also a clear difference between the axons of the two groups. The axons of the TRANSPLANT group atrophied (Figure 5, arrow + heart in A2), whereas those of the TRANSPLANT+PEG group were healthy (Figure 5, arrow + heart in B2).

### 3.4 The myelin of the TRANSPLANT group and the TRANSPLANT+PEG group were significantly different observed by scanning electron microscopy

**FIGURE 6:**
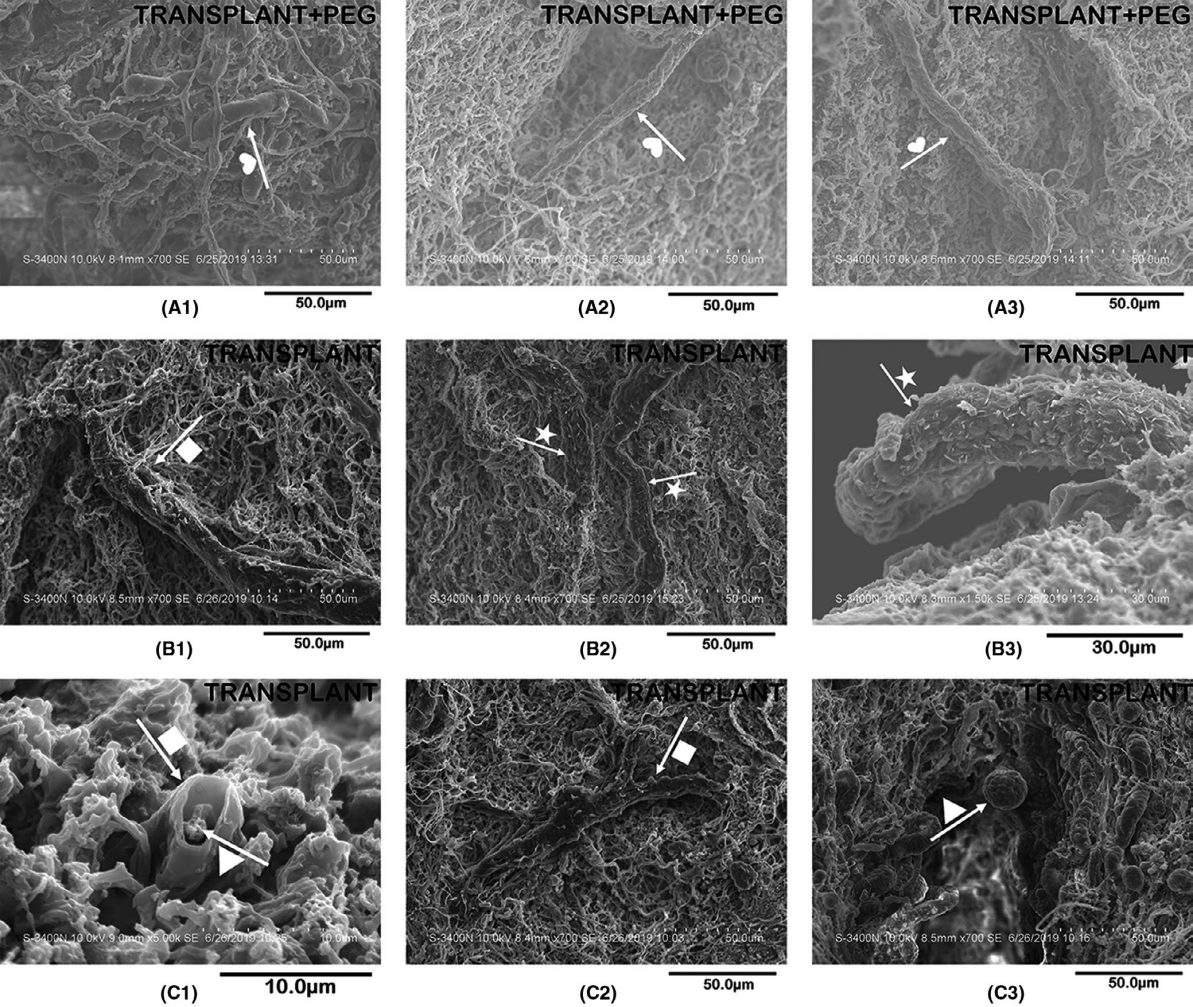
Scanning electron microscopy. In the bridging tissue of the TRANSPLANT group, most of the myelin is damaged (arrow + star in B2 and B3) and incomplete (arrow + square in B1 and C2). By comparison, the myelin of the TRANSPLANT+PEG group has a smooth surface and a complete structure (arrow + heart in A1, A2 and A3). At the edge of the sample blocks in the TRANSPLANT group, we find degenerated myelin (arrow + square in C1) enveloping a degenerated axon (arrow + triangle in C1, similar to the results in Figure 5, namely arrow + heart in A2). In the scars of the TRANSPLANT group, we also find axons with a spherically enlarged stump after degeneration (arrow + triangle in C3)

Changes in myelin and axons can be observed in more detail via scanning electron microscopy. In the scars of the TRANSPLANT group at the site of transection, most of the observed myelin was damaged (Figure 6, arrow + star in B2 and B3) and incomplete (Figure 6, arrow + square in B1 and C2). In comparison, the myelin of the TRANSPLANT+PEG group had a smooth surface and a complete structure (Figure 6, arrow + heart in A1, A2 and A3). In addition, the myelin from the TRANSPLANT+PEG group was generally thinner, suggesting that there was a smaller volume of new myelin. At the edge of the sample block (the position of the knife cut when sampling) in the TRANSPLANT group, degenerated myelin (Figure 6, arrow + square in C1) was seen to envelope a degenerated axon (Figure 6, arrow + triangle in C1), similar to that seen in Figure 5 (arrow + heart in A2). In the glial scars of the TRANSPLANT group, axons with spherical enlarged stumps after degeneration were also found (Figure 6, arrow + triangle in C3), which represent another sign of axonal degeneration.

### 3.5 Evidence of nerve recovery showed by histologic assessment

**FIGURE 7:**
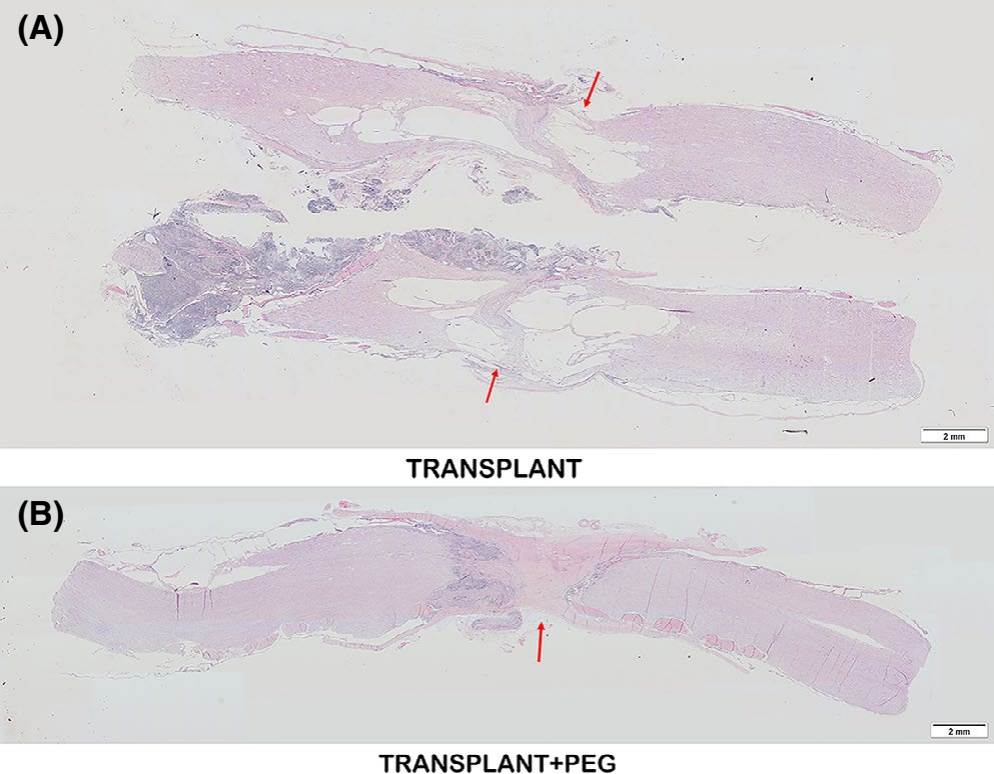
HE staining. Vacuolization due to tissue necrosis (cysts) is minimal in the TRANSPLANT+PEG group (red arrow in B), which is notably different from that of the TRANSPLANT group (red arrow in A)

**FIGURE 8:**
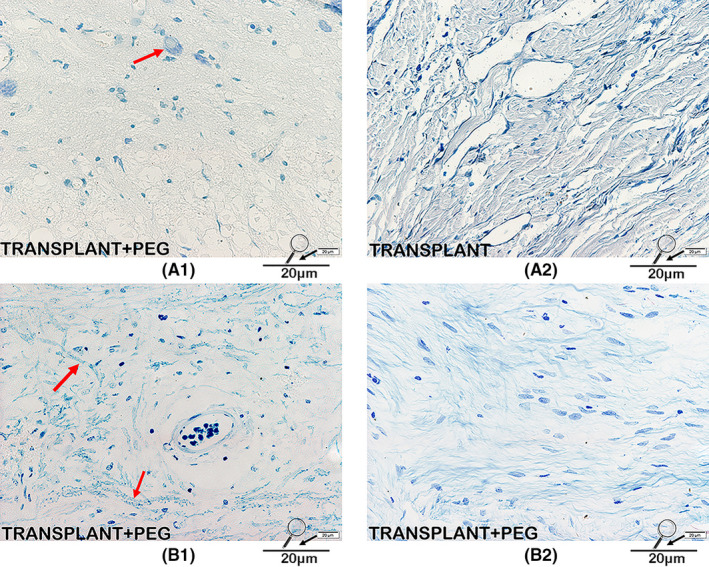
Nissl/LFB staining. Neurons labeled with Nissl bodies can be seen in the bridging tissue of the TRANSPLANT+PEG group (red arrow in A1) but not in the TRANSPLANT group (A2). On LFB staining, irregularly arranged myelin can be seen in the bridging tissue of the TRANSPLANT+PEG group (red arrows in B1), suggesting regrowth, while in the TRANSPLANT group, only stained myelin debris can be seen (B2)

In HE‐stained sections, the PEG‐treated and untreated spinal cords differed tremendously. In contrast to that of the TRANSPLANT group, vacuolization due to tissue injury was minimal in the TRANSPLANT+PEG group (Figure 7, red arrows in A and B), which likely demonstrates the neuroprotective effects of PEG. In terms of Nissl staining, neurons labeled with Nissl bodies could be seen only in the bridging of the TRANSPLANT+PEG group (Figure 8, red arrow in A1) but not the TRANSPLANT group (Figure 8, A2). After LFB staining, irregularly arranged myelin was found in the bridging tissue of the TRANSPLANT+PEG group (Figure 8, red arrow in B1), but only stained myelin debris was seen in the TRANSPLANT group (Figure 8, B2).

### 3.6 A large amount of MBP and neurofilament protein exist in the bridging tissue found by Immunohistochemistry

**FIGURE 9:**
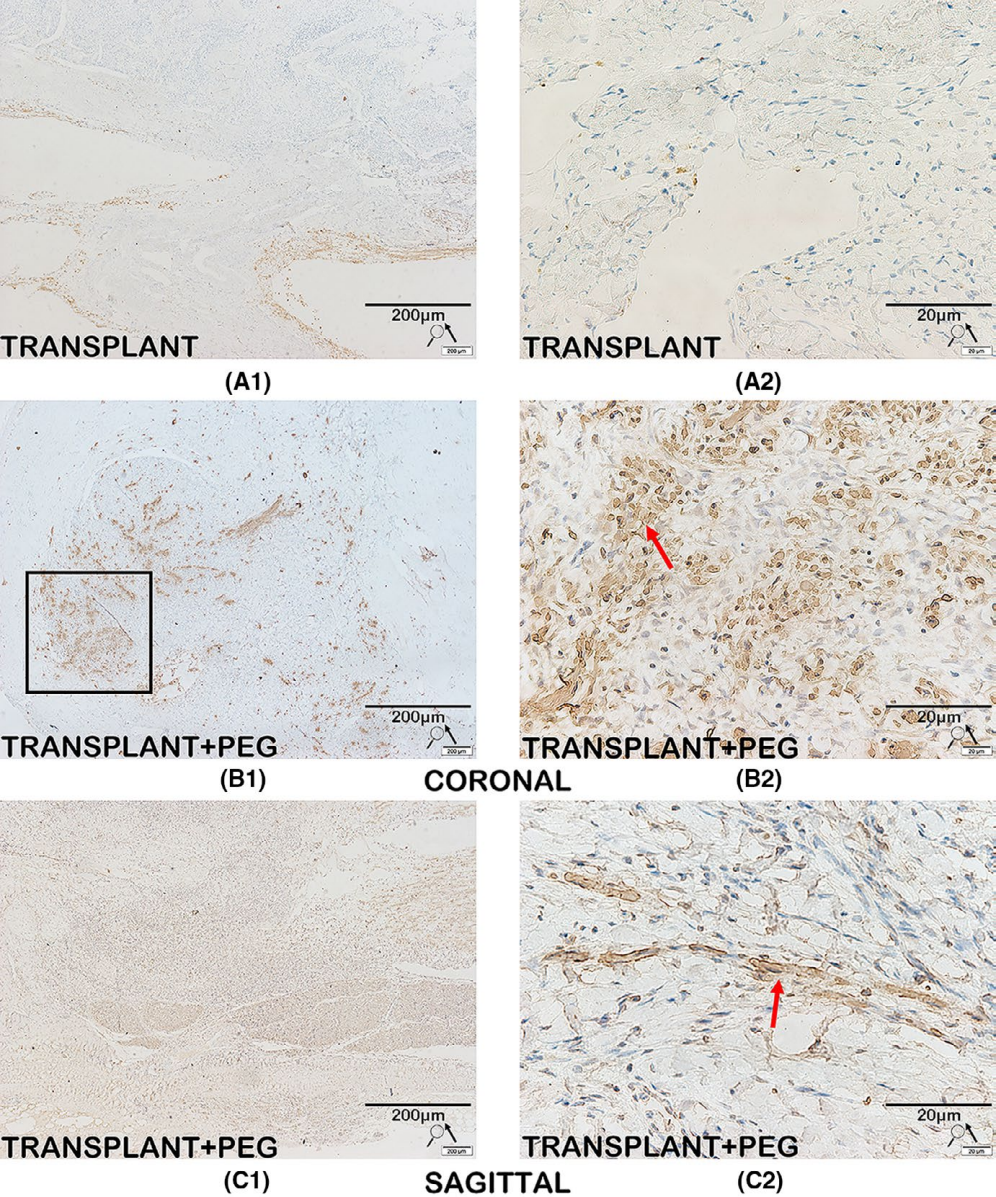
Immunohistochemistry for MBP. MBP is a major component and established marker of CNS myelin. There is a large amount of MBP in the bridging tissue of the TRANSPLANT+PEG group (B1, C1 and red arrows in B2, C2), while MBP is rarely seen in the TRANSPLANT group (A1 and A2). Surprisingly, we find a highly concentrated area of MBP in the representative coronal slice; this may represent the transplanted spinal cord (black square in B1)

**FIGURE 10:**
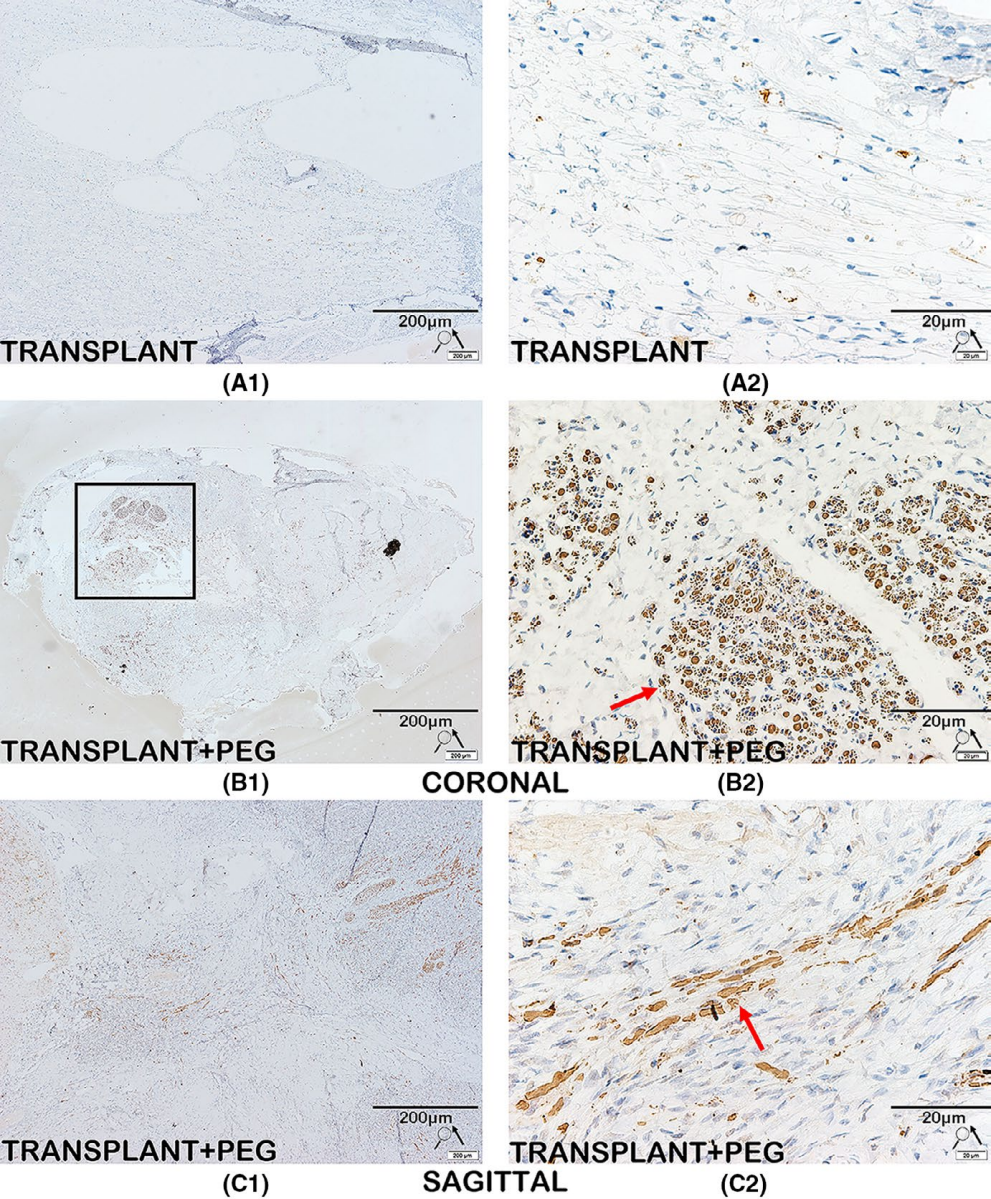
Immunohistochemistry for neurofilament protein. Immunolabeling of neurofilament protein is seen in the TRANSPLANT+PEG group (B1, C1 and red arrows in B2, C2), but not in the TRANSPLANT group (A1 and A2). We also find areas with high concentrations of axons in coronal slices (black square in B1); this is a sign of the survival of the transplanted spinal cord

CNS myelin contains a large proportion of MBP; thus, MBP is a well‐known marker for CNS myelin.^27^ A large quantity of MBP was observed in the bridging tissue of the TRANSPLANT+PEG group (Figure 9, B1, C1 and red arrows in B2, C2), but was seen only rarely in the TRANSPLANT group (Figure 9, A1 and A2). Surprisingly, a highly concentrated area of MBP was observed in the transverse section of the TRANSPLANT+PEG group (from the bridging tissue), which may have represented the transplanted spinal cord (Figure 9, black square in B1). However, in the TRANSPLANT group, no highly concentrated area of MBP was found. Semi‐quantitative analysis of AIODs revealed that there was a significant difference between the TRANSPLANT and TRANSPLANT+PEG groups (*p* = 0.0082). The presence of neurofilament protein, a critical component of the axonal cytoskeleton, indicated noticeable axonal sprouting across the transection site in the TRANSPLANT+PEG group (Figure 10, B1, C1 and red arrows in B2, C2), but not in the TRANSPLANT group (Figure 10, A1 and A2). There were also areas with high concentrations of axons in the transverse sections in the TRANSPLANT+PEG group (Figure 10, black square in B1) (from the bridging tissue), which are a sign of the viability of the transplanted segment of the spinal cord. The semi‐quantitative analysis of AIODs of neurofilament protein was also significantly different between the two groups (*p* = 0.0052).

### 3.7 Axons and myelin pass through the bridging tissue detected by Immunofluorescence

**FIGURE 11:**
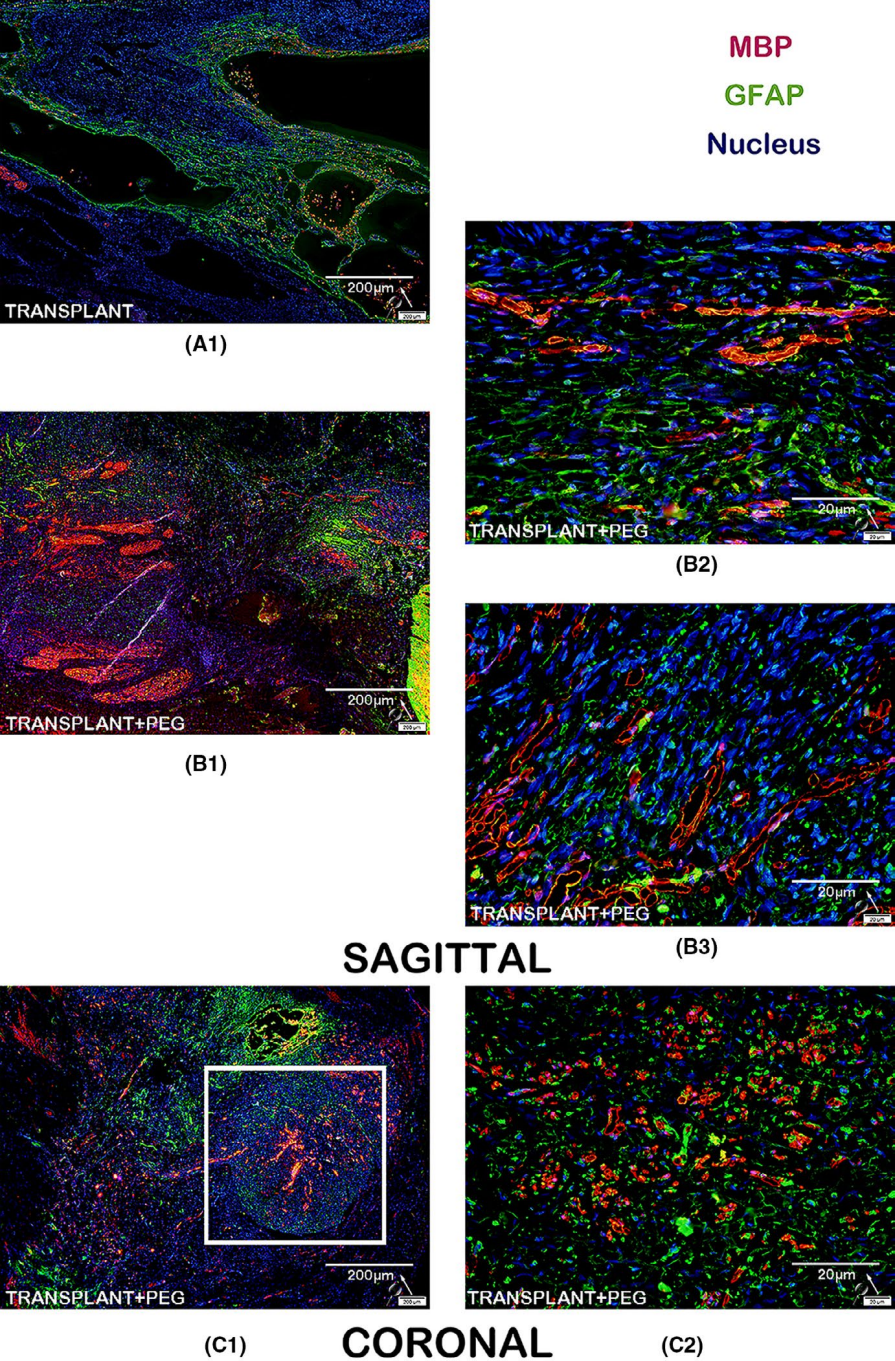
Immunofluorescence for MBP and GFAP. In the bridging tissue of the TRANSPLANT+PEG group, we observe a large amount of MBP (B1 and C1), but we did not find any MBP in the bridging tissue of the TRANSPLANT group (A1). In the magnified view, we can see that the MBP is intact (B2, B3 and C2). In the coronal view, there is dense myelin (white square in C1)

**FIGURE 12:**
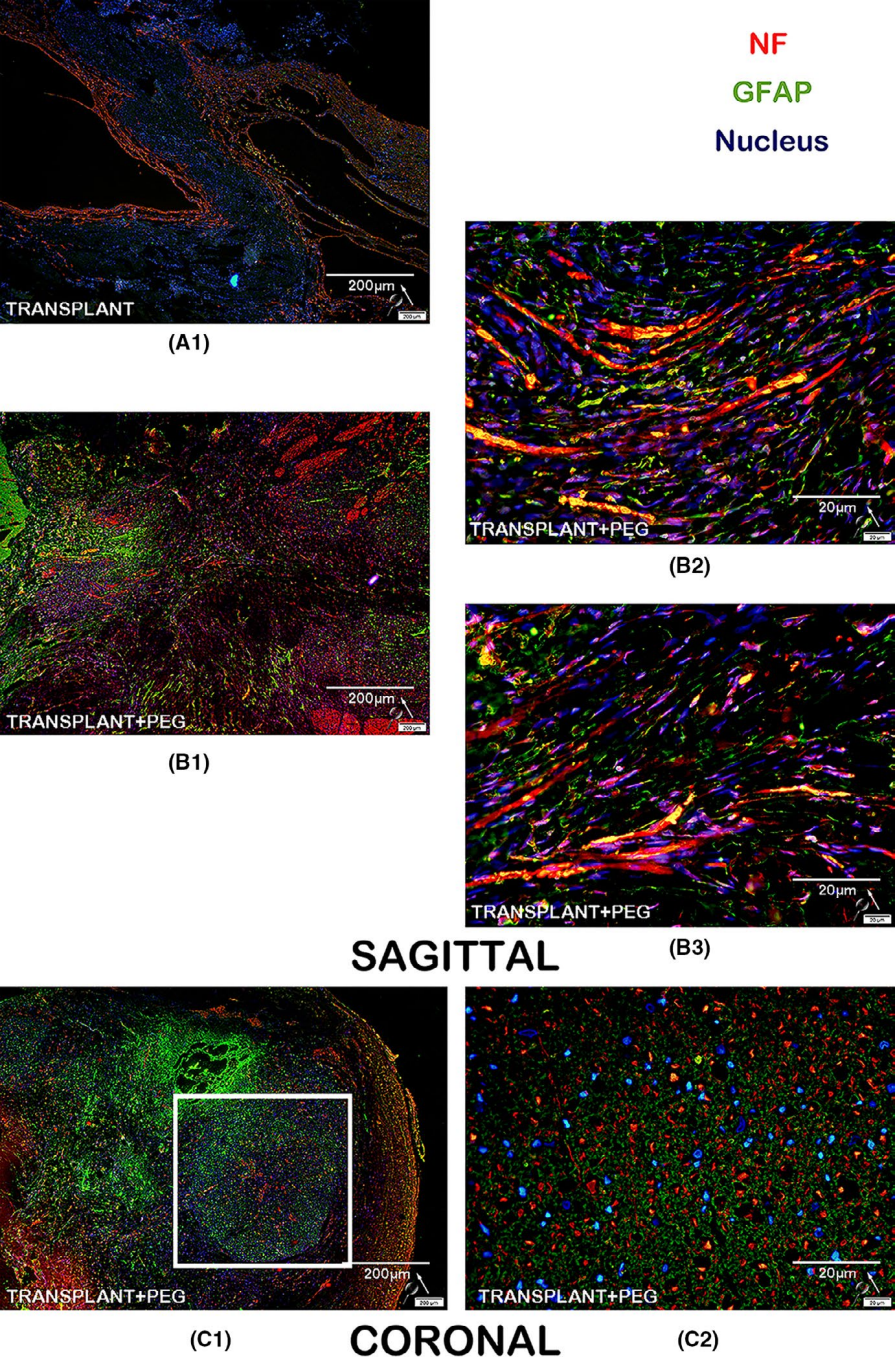
Immunofluorescence for neurofilament protein and GFAP: in the bridging tissue of the TRANSPLANT+PEG group, we observe a large amount of NF immunofluorescence indicative of neuronal fibers (B1 and C1), but we did not find similar immunofluorescence in the bridging tissue of the TRANSPLANT group (A1). In the magnified view, many apparent axons penetrate through the bridging tissue (B2, B3 and C2). In the coronal view, there is dense myelin (white square in C1)

When the immunofluorescent slides were assessed, we found that myelin was present in the bridging tissue of the TRANSPLANT+PEG group (Figure 11, B1, B2, B3, C1, and C2), but not in the TRANSPLANT group (Figure 11, A1). On one representative section, one can see a complete axon appearing to pass through the bridging tissue (Figure 11, B2). In the transverse section (from the bridging tissue), dense myelin was present (Figure 11, white square in C1). Neurofilament protein immunofluorescence identified that a large number of axons had passed through the bridging tissue area in the TRANSPLANT+PEG group (Figure 12, B1, B2, B3, C1, and C2), but not in the TRANSPLANT group (Figure 12, A1). Fibrotic scars and glial scars together built astroglial‐fibrotic scars, which is composed of fibronectin (FN)‐positive lesion core surrounded by a glial fibrillary acidic protein (GFAP)‐positive lesion border.^28^ The immunofluorescence identified that GFAP protein was present in the bridging tissue of the TRANSPLANT+PEG group and the TRANSPLANT group. This indicates that PEG may not have a significant inhibitory effect on the growth of glial scars, but it may have an inhibitory effect on fibrotic scars, which ultimately inhibits the growth of astroglial‐fibrotic scars.

## 4 DISCUSSION

There are at least four barriers to the effective treatment of SCI that must be overcome, as follows.^29^ First, amelioration/prevention of primary and secondary tissue damage at the SCI site is needed. CNS injury triggers microglial activation within minutes, constitute the innate immunity responsible for debris clearing and providing a source of trophic and antiinflammatory factors to promote tissue repair, but they also release inflammatory cytokines to fuel secondary injury,^30^, ^31^, ^32^, ^33^ inflammation is triggered and induces the release a wide variety of cytokines, chemokines, and other inflammatory mediators which can reduce nerve cell survival.^34^, ^35^ These destructive phenomena begin approximately 2 h after the injury and continue until about 6 months after the lesion.^36^ Second, acceleration and promotion of functional regeneration of long motor and sensory fibers are required. Abundant, long‐distance axonal regeneration may improve function distal to the lesion,^37^, ^38^ but there are many factors that hinder the regeneration of axons, such as the formation of an inhibitory extracellular matrix (glial scar) at the site of injury,^39^, ^40^ inhibitory myelin‐associated proteins,^41^, ^42^ and insufficient growth‐promoting factors.^43^ Third, restoration of sufficient myelination to damaged and regenerating axons is needed because the normal functioning of the nervous system requires the presence of myelin, which allows for rapid and efficient propagation of action potentials.^44^ Fourth, restoration of electrical continuity of axons and dendrites across the damaged SCI area is required. Astroglial‐fibrotic scar formation following spinal cord injury (SCI) is viewed as major obstacles that hinder axonal regeneration and functional recovery.^45^, ^46^, ^47^

At present, most studies on SCI have focused on these four issues. While research into SCI has made progress in the past decade, translating SCI research into clinical practice remains a challenge.^48^ Beyond rehabilitation, there has not been any approved SCI treatment designed to restore electrical continuity, so there is no treatment to restore central nervous system (CNS) control of motor and sensory function distal to the site of SCI.^49^ This failure may be partially because SCI patients usually have mature scar tissue in the spinal cord at the site of SCI, with varying degrees of necrosis and a lack of regenerative potential at both ends of the scar. Therefore, the primary focus of SCI treatment is in removing scar tissue and reconnecting the conduction pathway before the onset of the SCI‐induced inflammatory response and resultant formation of scar tissue.^50^ For example, anecdotal evidence from two patients with paraplegia showed that removing the necrotic segment of the spinal cord, using collagen or peripheral nerves to bridge the gap, led to a partial recovery of motor function after 1 year.^51^, ^52^

We and others have shown this dramatic re‐establishment of electrical continuity after spinal cord transection using PEG as a neuroprotective agent at the site of transection.^10^, ^14^, ^15^, ^16^, ^17^, ^18^, ^19^ However, clinically, spinal cord transection injury is not common, and paraplegic patients usually have a length of scar on the spinal cord. Through DTI examination, it was found that no fiber bundles passed through this scarred section of spinal cord, and the spinal cord at both ends of the scarred section was healthy spinal cord with fiber bundles. In the surgical treatment of human paraplegia, removing this scarred spinal cord section is very important to the treatment of SCI. However, if this scar tissue is removed, the continuity of the spinal cord will be interrupted, and a gap will be created at the location of the removed scarred spinal cord. To address this, we designed the following model: transplantation of a vascularized pedicle of hemisected spinal cord to establish spinal cord continuity after removal of a segment. This model created the same scenario as the spinal cord with fiber bundles at both ends after clinical removal of a section of scarred spinal cord from paraplegic patients.

This canine model of excision of a 1‐cm segment of the spinal cord with transplantation of a vascular pedicled hemisection from the immediately caudal part of the transected spinal cord to establish physical continuity after SCI. This proof‐of‐principle study in canines showed that paralysis after the removal of a 1‐cm‐long segment of the spinal cord can at least be partially reversed by a vascularized pedicle autograft of the distal hemicord to bridge the transected spinal cord after treatment with PEG at the contact interfaces according to the GEMINI SCF protocol.^7^, ^8^ The success of this model provides a new way to treat human paraplegics in the future.

Six months after operations and PEG treatment, the final average cBBB score in our present study was consistent with findings from our previous canine study of reinnervation of a completely transected spinal cord treated with PEG (median scores: 11.5 and 11).^17^ We used Mixed‐effects analysis (*α* = 0.05) to compare the effects of time and surgical methods (transplant+PEG or transection+PEG) on behavioral recovery, as reported previously.^34^ Through the mixed‐effects analysis of the transection+PEG group and the transplantation+PEG group (Table 2,b), the *p* value and *F* value of the factor ‘time’ were found to be <0.0001 and 114.6, respectively; thus, time has an impact on the recovery of spinal cord injury. The *p* value and *F* value of the factor ‘treatment (transplant+PEG group or transection+PEG group)’ were 0.6312 and 0.2469, respectively, so there was no significant difference between the transplant+PEG group and transection+PEG group. This new method replicates the results of previous experiments, and at the same time provides a new feasible method for future clinical operations. The main differences were a somewhat slower onset of symptom reversal in the present study (17 vs. 3 days) and greater final top scores in the previous study (12 vs. 18). These findings are likely due to the need for regrowth of fibers to cross two areas, instead of one area of spinal cord.

It is well known that recovery from SCI entails massive reorganization throughout the CNS.^26^, ^53^ The present remedial construct obviously required more intense and prolonged neuroplastic rearrangement than that of a simple transection. This finding lends support to another possible approach espoused originally by Freeman et al. in the 1960s, involving complete extirpation of the SCI epicenter, spinal shortening, and apposition of the freshly sectioned cord ends.^7^, ^54^ Using the hypothetical approach of Freeman, however, requires the need to remove one vertebra en bloc, which adds to the burden of surgery and may increase morbidity and mortality. Another difference concerns the minimal retraction gap that was found to follow full transection in previous studies.^14^, ^17^ In the present study, no gap remained, although potential between‐end compression may have created disturbances in local blood flow.

Diffusion tensor imaging and histologic data showed a vigorous regrowth of fibers across the sites of spinal cord fusion and re‐approximation in our present study. The GEMINI SCF protocol is predicated on propriospinal neurons of the gray matter sprouting fibers after the apposition of spinal cord surfaces under PEG protection, rather than on the regrowth of long pyramidal axons.^7^, ^55^ After the short fibers in the gray matter re‐connect, they bridge long‐range fibers (i.e., the pyramidal tract) on both sides, thereby restoring electrical continuity. This finding was confirmed in the present study. Notably, sphincter control did not recover, which is controlled by long‐range fibers, which apparently did not regrow or fuse at the site of the autograft.

Before the translation of this present research into clinical practice, several points should be noted. First, the hemicord to potentially be used as the bridge in patients with a chronic SCI will have undergone years of some form of plastic adaptive changes, some of which may be detrimental.^56^ These changes might interfere with the process of reinnervation in some situations or different types of injury; however, it is well known that so‐called entrenched plasticity is reversible.^57^ Second, nerve roots from the hemi‐graft have to be cut, which causes sensorimotor disruptions in their corresponding territories of original innervation. Studies of dorsal rhizotomies in patients with chronic pain have verified that nearby roots can compensate for such resulting deficits.^58^ However, the chest and abdominal wall muscles in this limited area would still be affected by motor deficits. Third, motor recovery and evidence of fiber regrowth indirectly confirmed that the graft retained its vascular inflow in the present study. However, spinal angiography should be a consideration in clinical trials. Finally, the use of PEG alone is associated with an impressive degree of recovery following spinal transection, but it is likely boosted by stimulation from the spinal cord and motor cortex, as per the GEMINI SCF protocol.^7^, ^55^

## 5 CONCLUSION

In summary, our present study provides the first evidence of reversal of motor paralysis after removal of a complete segment of the spinal cord and immediate bridging with an autologous vascular pedicle of the ventral hemisected spinal cord and PEG treatment (per the GEMINI SCF protocol). If these findings are confirmed, this phenomenon may lead to a novel and viable strategy for the treatment of selected patients with traumatic spinal paralysis.

## CONFLICT OF INTEREST

The authors declare that they have no conflict of interest.

## Data Availability

The data that support the findings of this study are available from the corresponding author upon reasonable request.
